# Optimizing drug selection from a prescription trajectory of one patient

**DOI:** 10.1038/s41746-021-00522-4

**Published:** 2021-10-20

**Authors:** Alejandro Aguayo-Orozco, Amalie Dahl Haue, Isabella Friis Jørgensen, David Westergaard, Pope Lloyd Moseley, Laust Hvas Mortensen, Søren Brunak

**Affiliations:** 1 Novo Nordisk Foundation Center for Protein Research, Faculty of Health and Medical Sciences, University of Copenhagen, 2200 Copenhagen, Denmark; 2 Statistics Denmark, 2100 Copenhagen, Denmark; 3 The Heart Center, Righospitalet, Copenhagen University Hospital, Copenhagen, Denmark; 4 Department of Obstetrics and Gynaecology, Amager Hvidovre Hospital, Copenhagen University Hospital, Hvidovre, Denmark; 5 Section of Epidemiology, Department of Public Health, University of Copenhagen, 1014 Copenhagen, Denmark

**Keywords:** Drug therapy, Drug regulation, Adverse effects, Epidemiology, Hypertension

## Abstract

It is unknown how sequential drug patterns convey information on a patient’s health status and treatment guidelines rarely account for this. Drug-agnostic longitudinal analyses of prescription trajectories in a population-wide setting are needed. In this cohort study, we used 24 years of data (1.1 billion prescriptions) from the Danish prescription registry to model the risk of sequentially redeeming a drug after another. Drug pairs were used to build multistep longitudinal prescription trajectories. These were subsequently used to stratify patients and calculate survival hazard ratios between the stratified groups. The similarity between prescription histories was used to determine individuals’ best treatment option. Over the course of 122 million person-years of observation, we identified 9 million common prescription trajectories and demonstrated their predictive power using hypertension as a case. Among patients treated with agents acting on the renin-angiotensin system we identified four groups: patients prescribed angiotensin converting enzyme (ACE) inhibitor without change, angiotensin receptor blockers (ARBs) without change, ACE with posterior change to ARB, and ARB posteriorly changed to ACE. In an adjusted time-to-event analysis, individuals treated with ACE compared to those treated with ARB had lower survival probability (hazard ratio, 0.73 [95% CI, 0.64–0.82]; *P* < 1 × 10^−16^). Replication in UK Biobank data showed the same trends. Prescription trajectories can provide novel insights into how individuals’ drug use change over time, identify suboptimal or futile prescriptions and suggest initial treatments different from first line therapies. Observations of this kind may also be important when updating treatment guidelines.

## Introduction

High cost of prescription drugs has become a problem in most Western societies, where the expenditure of prescription is ever increasing and consumes a growing share of the total healthcare outlay^1,2^. A better understanding of variation in treatment response and tolerance would increase quality of life and improve treatment guidelines while also reducing costs of maintaining a healthy ageing population partly via prescribed treatment^3^. During the past 40 years, handwritten prescriptions have largely been replaced by electronic versions, and in most Western countries e-health infrastructures have been designed to manage them^4^. This transformation has facilitated secondary use of healthcare data, including studies of prescription databases. Since 1995, all prescriptions redeemed at a pharmacy in Denmark have been registered in the Danish National Prescription Registry (DNPR), now holding data on 7.2 million people^5^. DNPR is among the oldest and largest prescription registries in the world. Examples of other prescription data resources include the Finnish database on drug utilization that was stablished in 1994^6^, and the UK Clinical Practice Research Datalink (CPRD), which also holds prescription data^7^.

Research using these resources has been performed mostly in a hypothesis driven manner and highly targeted, either in terms of diseases (e.g., myocardial infarction)^8^ or drugs (e.g., antibiotics and gastric acid inhibitors)^9–11^. In a large longitudinal study, prescriptions from a cohort of over 9 million people were used to conclude that 26% of all prescribed drugs influence cancer risk^12^. Other studies have addressed opioid use and the rapid increase in opioid prescription over the last decades^13^. Prescription studies have also been designed to predict prescriptions based on previous, redeemed prescriptions. Data-mining prescriptions of anti-diabetic drugs have, for example, been used to predict future anti-diabetic prescription^14^. These studies involve less than one million people and focus on a subset of all drugs only^15,16^.

Numerous guidelines derived from randomized controlled trials (RCTs) and meta-analyses—for specific diseases and complications—have been published, e.g., hypertension, diabetes, and cardiovascular complications^17^. However, such guidelines rarely consider the full spectrum of diseases that are often being treated simultaneously; and typically, guidelines present an overview of state-of-the-art literature and evidence levels rather than answers to conflicting clinical considerations. This traditional limitation may, in turn, result in inadequate or unnecessary treatment for some patients^18^. Yet, the digital transformation, where entire health registries are being studied in a data-driven manner, can potentially compliment results from classical RCTs and meta-analyses in understanding the variation in treatment responses and tolerance^19^.

Previous longitudinal analyses of sequential drug prescriptions have been performed in a targeted fashion based on selected diseases or groups of drugs. However, it is unknown how sequential drugs patterns convey information about an individual’s health status and treatment guidelines rarely account for this. This study is the first to perform a drug-agnostic longitudinal analysis of prescription trajectories in a population-wide setting.

The objective of this cohort study was to identify and characterize the most prevalent prescription trajectories in the Danish population, hence providing a model to help fill gaps between disease registry data from hospitals, indications from general practitioners and dispensed prescriptions.

Mapping population-wide prescription trajectories can provide novel insight into how individuals’ drug use change over time. This method can—at the level of individuals—identify potentially suboptimal prescription sequences, as well as futile prescriptions.

## Results

### Condensing prescription data into trajectories

We performed a comprehensive cohort analysis of DNPR covering prescription patterns across all drugs dispensed at all pharmacies in a nation-wide, universal healthcare setting. We condensed the longitudinal prescription redemption data on each individual into prescription trajectories. Then we characterized over 9 million prescription trajectories of varying lengths among 7,255,919 individuals, 49.3% male and 50.7% female, (Supplementary Tables 1 and 2). For 48% of the population (3.5 million people) their presence in the registry start in 1995 and end in 2019 (Supplementary Figs. 1–3).

**Fig. 1:**
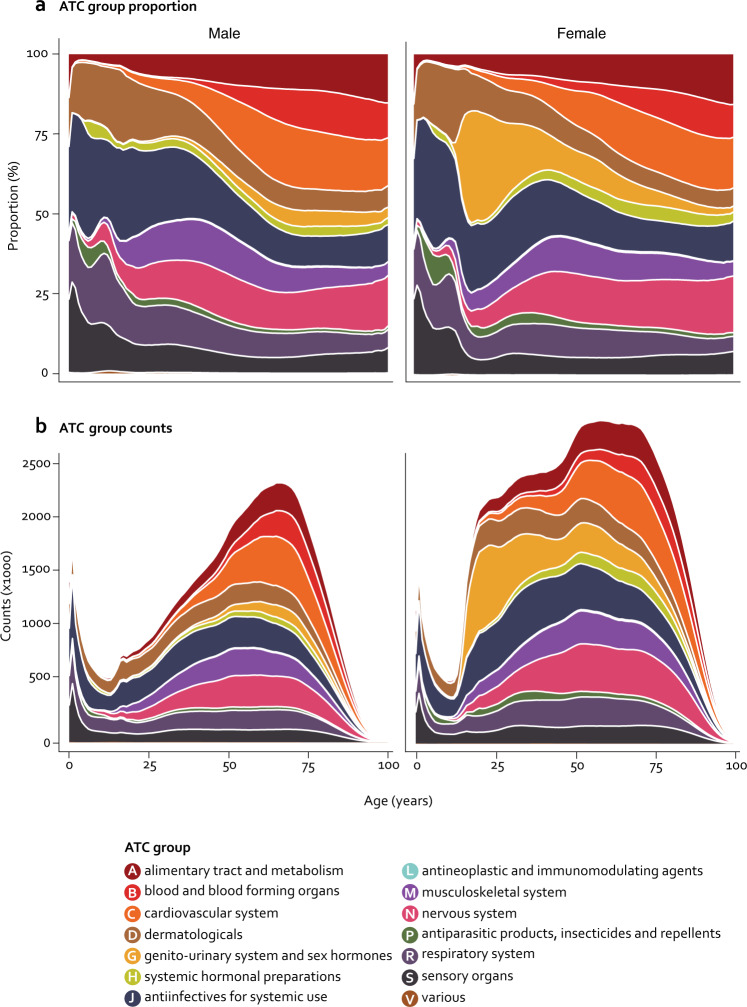
Prescriptions redeemed at all Danish pharmacies in the period 1995-2019 stratified by the 14 anatomical ATC groups for males (left) and females (right). **a** Proportion of different ATC groups against age at redemption. **b** Total counts of prescriptions against age at redemption. Color key according to anatomical ATC group.

In Fig. 1a females display an increase in the proportion of anatomical group G (genitourinary system and sex hormones) during the teenage period. Furthermore, for both sexes in the age interval 5–15 years there is an increase in the proportion of anatomical groups H and P (systemic hormonal preparations, excluding sex hormones and insulins; and antiparasitic products, insecticides and repellents), possibly related to growth and the infections of childhood. At older ages, the proportions of drugs related to anatomical group B and C (blood forming organs and cardiovascular system) increase, representing medication for the most common diseases of the age group such as hypertension and atherosclerosis (Fig. 1a). Figure 1b shows a spike at young age for both sexes, primarily related to anatomical groups J, R, and S (anti-infectives for systemic use, respiratory system, and sensory organs). There is also a continuous increase from young adults with a peak value at the age of 65–70 years for males and 75 years in females, reflecting that the prevalence of polypharmacy increases with age and that life expectancy is higher for females than for males (Supplementary Fig. 4).

### Temporal drug prescription association analysis

From the full data set, 229,803 sequential drug pairs (P1→P2) were identified, where all chemical subgroups of each anatomical group were represented. We then excluded all prescription pairs that were redeemed by less than 100 patients, those with *Q-*value above 10^−03^ and those with RR < 1 (Supplementary Fig. 4a).

**Fig. 2:**
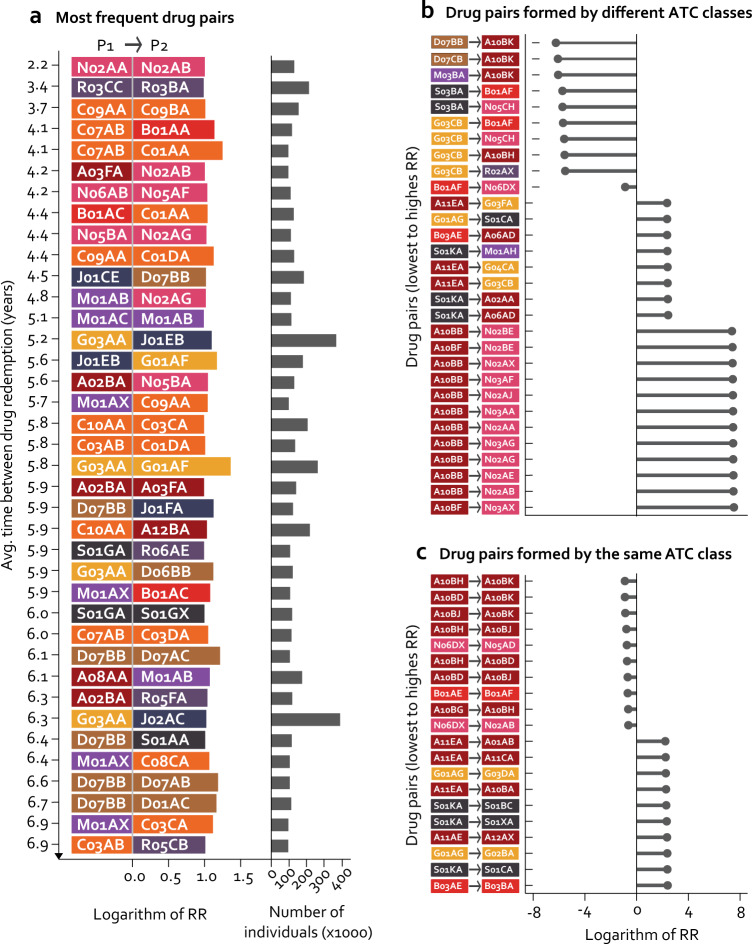
Selected directional prescription pairs. **a** Attrition of prescription pairs following temporal statistical modeling. This includes the drug pair steps (P1 → P2) that were taken sequentially by over 100,000 individuals, had a relative risk ratio (RR) > 2 and a *Q*-value < 10^−03^. Drug pairs were ordered vertically by time between P1 and P2 from shortest to longest time (top to bottom). Sizes of bars for each prescription (P1 and P2) are equal and correspond to the absolute number of the logarithm of its RR. The pair with the largest RR is G03AA→G01AF. The unannotated bars to the right indicate the number of individuals following each of the directional prescription pairs. **b** Drug pairs (P1→P2) with the lowest and highest RR where P1 and P2 belong different ATC classes. **c** Drug pairs (P1→P2) with the lowest and highest RR where P1 and P2 belong to the same ATC class. Pairs are ordered by RR, lowest to highest (top to bottom). *Refer to Fig. 1 for ATC group legend.

Figure 2a (Supplementary Table 3) presents the most frequent, statistically significant directional prescription pairs (classified by chemical ATC class). All pairs were prescribed to more than 100,000 people and interestingly all had an RR > 2. The pair with shortest average intermediate time is formed by two drugs that belong to the nervous system group N02AA→N02AB, natural opium alkaloids and phenylpiperidine derivatives, respectively (RR = 2.76, 95% confidence interval (95% CI): 2.74–4.13, *P* = 2.70 × 10^−8^), with an average intermediate time of 2 years. Beta blocking agents redemption increases the risk for subsequent redemption of digitalis glycoside (C07AB →C01AA; RR = 3.55, 95% CI: 2.30–5.47, *P* = 9.07 × 10^−9^). Both chemical subgroups are used as antiarrhythmic drugs. The pair with the highest RR (~4) is G03AA→G01AF, progestogens and estrogens, which include hormonal contraceptives, to imidazole derivatives, respectively (RR = 3.98, 95% CI: 2.42–6.55, *P* = 5.27 × 10^−8^), while the pair with the highest number of patients is G03AA→J02AC, triazole derivatives (RR = 2.82, 95% CI: 1.76–4.53, *P* = 1.66 × 10^−5^). Both imidazole and triazole derivatives are used to treat fungal infections^20^.

The directional prescription pair of drugs from different anatomical groups having the lowest RR is D07BB→AB10K, corticosteroids, moderately potent, combination with antiseptics, and sodium-glucose co-transporter 2 (SGLT2) inhibitors, respectively (RR = 0.002, 95% CI: 0.001–0.003, *P* = 4.44 × 10^−142^). An increased risk of redemption of opioids (N02) is observed after prescription of diabetic treatment, particularly blood glucose lowering drugs (A10B). Diabetic patients often use opioids to treat neuropathic pain derived from diabetes^21^. The use of direct factor Xa inhibitors (B01AF), which is a group of antithrombotic agents associated with a lower risk of subsequent redemption of other anti-dementia drugs (N06DX) (RR = 0.41, 95% CI: 0.37–0.46, *P* = 1.38 × 10^−62^) (Fig. 2b). In Fig. 2c pairs that belong to the same anatomical group show that there is a high risk of taking vitamin B12 (B03BA) after redemption of iron (B03AE) (RR = 9.87, 95% CI: 6.78–14.37, *P* = 6.95 × 10^−33^). Patients redeeming GLP-1 prescriptions (A10BH) are at lower risk of redeeming SGLT-2 (A10BJ) prescriptions subsequently (RR = 0.45, 95% CI: 0.37–0.55, *P* = 1.34 × 10^−14^). Both types of medication are effective antihyperglycemics and have also been associated with cardiovascular protection^22^.

### Prescription trajectories across chemical subgroups

**Fig. 3:**
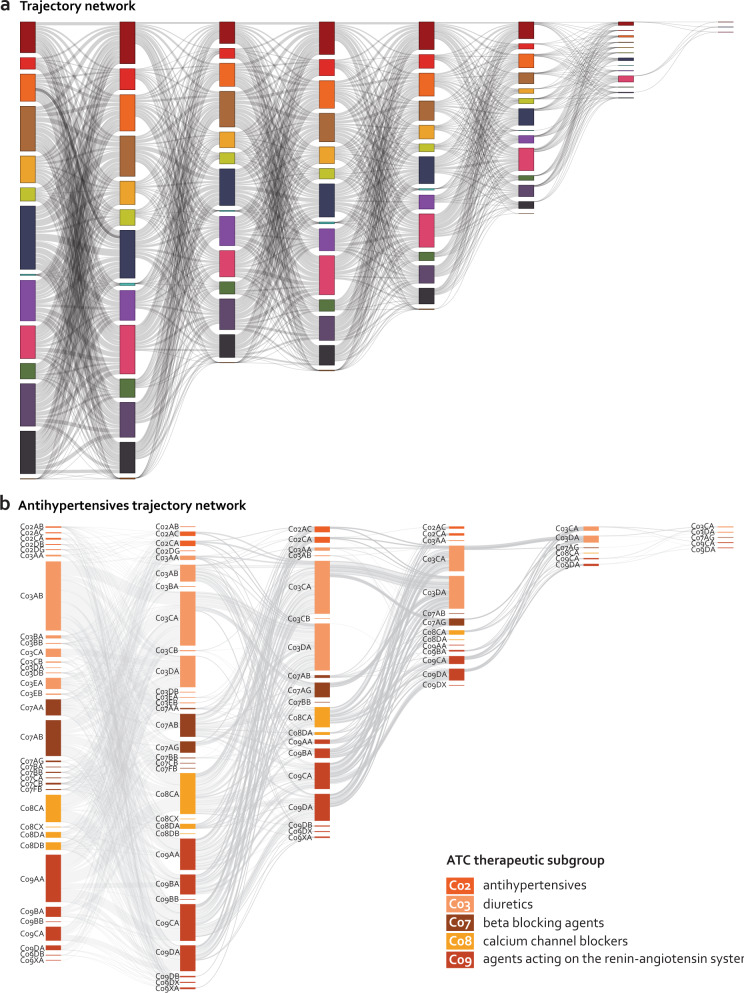
Prescription trajectory space. **a** All statistically significant prescription trajectories followed by more than 1000 patients are represented in this interconnected network. The order of the trajectories and the organization of the network is arbitrary. The length of the trajectory is depicted by the number of nodes it contains (from left to right, ranging from 2 to 8). The heterogeneity of prescription trajectories is depicted here. **b** Alluvial plot of the chemical subgroups used in treatment for hypertension, including C02, C03, C07, C08, C09 (antihypertensives, diuretics, beta blocking agents, calcium channel blockers, and agents acting on the renin-angiotensin system, respectively). The plot is ordered by length from left to right, and arbitrarily by chemical subgroup from top to bottom. The edge height represents the number of people that move from one node to the next (going left to right). Note that patients can follow more than one trajectory at the same time. *Refer to Fig. 1 for ATC group legend in.

The directional prescription pairs were then combined into prescription trajectories defined by recurrent prescriptions of three or more chemical subgroups, which were followed by a minimum of 1000 patients (Table 1). Trajectories containing prescriptions of four different chemical subgroups exceeded 3.2 million. All trajectories form a heterogeneous network with intertwined connections across anatomical subgroups (Fig. 3a), attesting to the complexity of prescription patterns, as well as the fine-grained level of patient stratification these analyses can support. Supplementary Fig. 4a displays trajectories where all prescriptions are classified in the same anatomical subgroup. The largest number of trajectories is formed by ATC groups J, D, and N (antiinfectives, dermatologicals, and nervous system, respectively).

**Table 1:** Quantitative summary of the number of prescription trajectories.

Trajectory length	Number of combinatorial possibilities (×10^9^)	Number of trajectories (followed by >1000 patients)	RR > 1*	Number of patients	Avg. time of trajectories (days)
2	0.00016	38,607	26,018	6,971,152	2,903
3	0.031	86,715	570,374	6,092,274	4,436
4	4.52	4,595,060	3,267,979	5,970,284	5,327
5	519.24	4,314,951	3,186,683	4,547,832	5,880
6	49,414.73	3,779,496	2,411,796	3,593,486	7,708
7	4,023,771.39	183,213	81,417	1,475,953	8,594
8	286,190,740.36	203	62	53,512	9,060

We found that shifts between different cardiovascular drugs are heterogeneous and complex (Fig. 3B). Patients starting with low-ceiling diuretics (C03AB) continue to another prescription within this ATC group in 77% of the cases, whereas 26% of patients starting with high-ceiling diuretics (C03CA) continue to another prescription in this ATC group (Supplementary Fig. 4b). The use of plain sulfonamides significantly increases the risk that the patient with this prescription later will be prescribed potassium-sparing agents (C03CA → C03DB; RR = 6.43, 95% CI: 4.16–9.93, *P* = 5.40 × 10^−17^). The use of thiazides and potassium in combination (C03AB) increases the risk of redeeming a prescription subsequently that comprises several other drug subgroups, such as beta-blocking agents (C07), dihydropyridine derivatives (C08CA), angiotensin II antagonists (C09C and C09D), among others (Fig. 3B).

### Using prescription trajectories in risk stratification

In Fig. 3b, the chemical subgroup agents acting on the renin-angiotensin system (C09) show a large number of trajectories interconnected across all chemical subgroups, following prescriptions within the same group subsequently. More than 50% of those who ever redeemed C09A (ACE inhibitors, plain) (*n* = 1,074,196) also eventually redeemed a second drug from the chemical subgroup C09 (n = 569,212). More than six thousand individuals (*n* = 6379) follow a trajectory that starts with C09A, followed by C09B (ACE inhibitors, combinations), C09C (angiotensin II receptor blockers (ARBs), plain) and finally C09D (angiotensin II receptor blockers (ARBs), combinations) in a short span of time (average 7 years between first and last redemption).

**Fig. 4:**
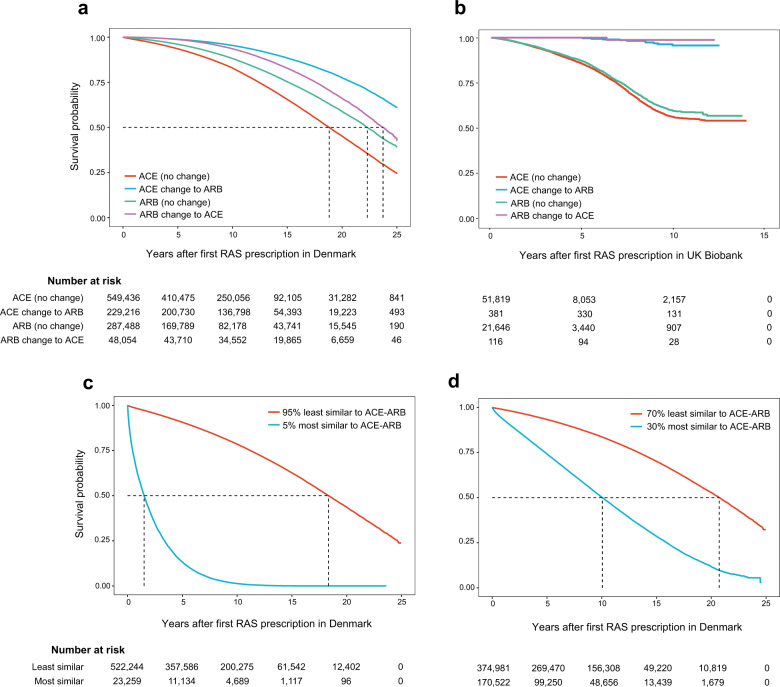
Survival curves for patients treated with different RAS drugs as different lines of treatment. **a** Survival curves for different risk groups stratified by prescription trajectories. Age, sex, and CCI adjusted Cox proportional hazard survival probability by groups of RAS treatment: i) redeeming solely ACE inhibitors with no change to ARB; ii) redeeming ACE inhibitors with posterior change to ARB; iii) redeeming uniquely ARB(s) with no change to ACE; and iv) redeeming ARB(s) with posterior change to ACE. The hazard ratio (HR) is lower for all individuals treated solely with ARB or treated with ARB before or after ACE when compared to individuals treated with ACE with no change to second line treatment. **b** Survival curves for the same groups of patients in the UKBB. **c** 95th percentile most similar ACE patients to AC–-ARB trajectories survival vs. ACE patients less similar to ACE–ARB. **d** Seventieth percentile most similar ACE patients to ACE–ARB trajectories survival vs. ACE patients less similar to ACE–ARB.

Next, we tested if the differences in proportion of patients starting with ACE inhibitors and ARBs, and subsequently redeeming another cardiovascular drug or not, would correlate with the 24-year survival probability of these patients. The demographics of patients treated with ACE inhibitors (C09A and C09B) and having no earlier C09 redemptions, and patients who posterior to ACE got prescribed ARBs (C09C and C09D) were similar (Supplementary Table 4). However, we identified four groups of patients with different survival probabilities (Fig. 4a). A Cox proportional hazard model showed that individuals treated solely with ACE are associated with an increased hazard of mortality. Individuals treated with ARBs as first line treatment and with no posterior change in prescription vs individuals treated only with ACE inhibitors have an HR < 1 (HR = 0.73, 95% CI: 0.64–0.82, *P* < 1 × 10^−16^). Individuals treated with ACE inhibitors as first line treatment with posterior change to ARBs vs individuals treated with ACE inhibitors, and individuals treated with ARBs with posterior change to ACE vs. individuals treated with ACE and no change, also have HR < 1 (HR = 0.47, 95% CI: 0.41–0.54, *P* < 1 × 10^−16^; and HR = 0.96, 95% CI: 0.77–1.19, *P* < 1 × 10^−16^, respectively).

We then used the UKB self-reported medication information to stratify patients according to RAS treatment, as described above. Similarly to the results obtained using the stratification by RAS of patients in DNPR, a Cox proportional hazard model showed that individuals treated solely with ACE inhibitors had a higher HR compared with the other three groups of patients (ARB vs. ACE: HR = 0.90, 95% CI: 0.84–0.95, *P* < 1 × 10^−3^; ACE–ARB vs. ACE: HR = 0.06, 95% CI: 0.03–0.11, *P* < 1 × 10^−16^; ARB–ACE vs. ACE: HR = 0.02, 95% CI: 0.003–0.16, *P* < 1 × 10^−4^). Interestingly, these groups had different survival probabilities (Fig. 4b).

Next, we calculated the trajectory similarity of patients treated with ACE with no change (ACE) to patients treated with ACE with posterior change to ARB (ACE–ARB), based on their length four trajectories leading up to RAS. Using the adjusted Tanimoto similarity score we ranked the patients treated with ACE based on similarity to each frequent trajectory (followed by more than 1000 patients) from ACE–ARB patients. The survival probability of those individuals, whose trajectory history leading to RAS was most similar to the trajectories of ACE–ARB was significantly lower. The top 5% (95th percentile) most similar patients had a survival probability loss of almost 15 years in comparison to the rest of the ACE patients; and the top 30% (70th percentile) most similar patients to ACE–ARB had a survival probability of approximately 10 years less compared to the rest of ACE patients (Fig. 4c, d).

## Discussion

This study sheds light on the immense heterogeneity in the prescription trajectory space in a population covered by a one-payer health model. As population-wide health research with registries and EHRs evolves rapidly, it grows closer to account for patient dissimilarities, i.e., advance precision medicine initiatives^23–27^.

Treatment decisions can be supported by these longitudinal analyses, as, for instance, the findings for increased risk after C03AB of different diuretic drug redemptions. Thus, prescribing both drugs at the same time, or changing the first line treatment for some groups of patients might improve the outcome and reduce the risk of multiple unnecessary treatment shifts. This finding is of special importance for anti-hypertensive drugs as more than 70% of the patients in this study that have a prescription for an anti-hypertensive will be prescribed more than one^28,29^.

Patients with hypertension are very heterogeneous and display different tolerance to drugs. Prescription trajectories might in many cases represent the existing treatment guidelines, which indicate the sequence of drugs to administer given specific diseases and symptoms, and only in case of side effects or incapacity to tackle the diseases can the doctor prescribe the subsequent drugs (primary, secondary, and third prophylaxis)^30^. As exemplified above, in more than 50% of cases, the use of ACE inhibitors (C90A and C09B) as a first prescribed drug is followed by a series of different drugs within the same therapeutic group (C09). Often, drugs redeemed after ACE inhibitors include C09C and C09D. These trajectories suggest that the treatment is more effective when reaching the level of ARB therapy in the guideline. International guidelines advise to consider ARBs as first-line antihypertensive therapy only if there is a compelling indication^31^; the present trajectory-based analysis offers an insight that perhaps most patients receive no substantial benefits from the first line of treatment and only when reaching later treatments, such as C09C or C09D are they are treated effectively. Individuals’ trajectory similarity can be used to support decision treatment in patients that require treatment with RAS drugs.

Our study had several strengths and limitations. Using registry data from the entire Danish population ensured a large sample size preventing problems related to inclusion and exclusion, as well as cohort grouping during the drug pair modeling. Furthermore, using a nation-wide registry with structured information on prescription redemptions, where redemptions are always registered, guaranteed that we did not miss many cases in our study. Nevertheless, codes will be missing in the cases where drugs are sold over the counter or dispensed directly by health care providers. Second, the prescription of drugs will be limited to the decision made by the physician, making observations confounded by indication. Third, with respect to the number of people that follow a trajectory, those formed by drugs commonly prescribed will be over-represented.

Some unaddressed challenges remain. For example, neither treatment compliance nor treatment duration is considered in the current version of the model. However, we argue that prevalent diseases like hypertension are ripe for analysis using this strategy given the large size of the data set and plethora of treatment options. We exemplify this using the prescription trajectories for ACE inhibitors and ARBs (Fig. 3). This model was developed based on the Danish population and thus a population with a homogenous demography and generally a low level of emigration, with 92.5% being European and the next most prevalent region of origin being Asia (2.9%) (Supplementary Table 5)^32^. The prescription trajectories might be confounded by patient characteristics. In order to reduce bias due to sex, age at redemption, or year of prescription, these were introduced as covariates in the model. For specific cases other covariates, such as specific diseases or other prescriptions, could be used. However, we did not include it in the general analysis as these are case-dependent, and we aimed for presenting a generic approach. Future work will be focused on integration of additional data sources, e.g., data regarding socio-economic status.

We identified the most prevalent prescription patterns in the Danish population, hence providing a model to help fill gaps between disease registry data from hospitals, indications from general practitioners and the dispensed prescription. Ultimately, the implementation of prescription trajectories in clinical decision support may aid in better patient stratification, further advancing modern healthcare towards personalized medicine.

## Methods

### Study design

Using the Danish Central Person Registry (CPR) number, a unique identifier to each individual implemented in 1968, all residents of Denmark can be tracked and followed over time^32^.

We identified a cohort of all individuals in the Danish population recorded in CPR between January 1st, 1995 and June 30th, 2019 (*n* = 7,873,901), who were at risk of redeeming one or more prescriptions. Prescription redemption is recorded in the population-based Danish National Prescription Registry (DNPR), and we found that 91% (*n* = 7,255,919) of the population at risk redeemed at least one prescription in the study period.

From the DNPR, we extracted features from the Anatomical Therapeutic Chemical (ATC) Classification System at the chemical subgroup level (i.e., the 4th ATC level). For each individual and each ATC chemical subgroup (e.g., A10BA, biguanides), only the first redemption was included in the analysis.

Information regarding diseases was obtained from the Danish National Patient Registry, which contains administrative information related to hospital admissions such as primary and secondary diagnoses, which uses the International Statistical Classification of Diseases and Related Health Problems 10th revision (ICD-10) during the period 1994 to December 2017.

The UK Biobank (UKB) self-reported medication data were used to replicate patient stratification and survival analysis results.

### Statistical analysis

We examined all sequential redeemed prescriptions, i.e. two prescriptions redeemed at two different times by the same individual, for all patients across a 24-year period. The relative risk ratio of redeeming P2 after P1 relative to those who did not redeem P1 was modeled using a Poisson regression model, where the patients’ counts were treated as the dependent variable. The analyses were adjusted for sex, age, calendar year, and time at risk as offset parameter. As patients are often observed over time, a single patient can contribute to multiple redemption, age and calendar year categories. Consequently, prescription redemption, age and calendar year were treated as time-varying factors in the analysis (see Supplementary Fig. 5 for an illustration).

To establish whether the parameters in the model are significant, a Wald Chi-squared test was used for each Poisson model to obtain associated *p*-values. *Q*-values were calculated from the *p*-values using the Benjamini–Hochberg (BH) procedure. Moreover, the event counts may have a variation greater than that predicted by a Poisson distribution, thus leading to overdispersion. Hence, some pairs will have too small *p*-values.

To determine directionality of the pairs (P1→P2 or P2→P1) we compare the RR derived from the model for each prescription co-occurrence. We used the *p*-value of both to select the more significant direction and we posteriorly run a binomial test to validate them.

Pairs with a significant directionality (P1→P2 or P2→P1) were iteratively joined in prescription trajectories to identify longitudinal trajectories containing three or more prescriptions. These were obtained by combining pairs with overlapping prescriptions (P1→P2 and P2→P3 combined to P1→P2→P3). They were subsequently extended with more overlapping prescription pairs to obtain longer trajectories. In order to add robustness to the trajectories, we selected only those followed by 1000 or more patients. The average time difference between each prescription pair in the trajectory was computed.

### Survival analysis

We evaluated the association between prescription pairs with a significant directionality and death for the individuals who had been exposed to renin-angiotensin agents (C09). Patients were included if they had been prescribed any C09 twice or more, at any point in their recorded history. Patients were followed up until death or end of data period, which was up to 24 years. This was modeled using a left-truncated right-censored multivariable Cox regression model with individuals contributing to the risk group from the time they redeem the first prescription of interest. The risk group was compared to the individuals who did not redeem any prescription in the C09 subgroup twice or more. The model included age, sex, and Charlson comorbidity index (CCI)^33^ as covariates (Supplementary Fig. 6).

### Trajectory similarity analysis

The similarity between redeemed, individual prescription histories was calculated using length four trajectories (followed by more than 1000 patients and up to 5 years before the first redeemed RAS drug). Patients treated with ACE inhibitors and no posterior change to ARB, where the last prescription was the RAS drug, were compared at the trajectory level to patients treated with ACE with posterior change to ARB (ACE–ARB). To quantify the similarity, we used an adjusted Tanimoto similarity score^34^. Each patient’s prescription history in terms of statistically significant drug pairs in their length four trajectories leading to the first RAS treatment were compared against each frequent trajectory followed by ACE–ARB patients. The intersection of prescriptions was divided by the total number, *PP*, of elements in the ACE–ARB trajectory,

1 $${\rm{sim}}\left( {{\rm{patient}},{\rm{ACE}} - {\rm{ARB}}} \right) = \frac{{\mathop {\sum }\nolimits_{k = 0}^n \frac{{|{\rm{PP}}\;{\rm{in}}\;{\rm{patient}}\;history \cap {\rm{PP}}\;{\rm{in}}\;{\rm{ACE}} - {\rm{ARB}}_{{\rm{trajectory}},{\rm{k}}}|}}{{|{\rm{PP}}\;{\rm{in}}\;{\rm{ACE}} - {\rm{ARB}}_{{\rm{trajectory}},{\rm{k}}}|}}}}{n}$$

Similarity analysis was performed across all ACE–ARB trajectories for each patient treated with ACE and no switching to other RAS drugs. The closer to 1 the more similar the patient is to the frequent trajectories followed by ACE–ARB patients. To quantify the overall similarity, we calculated the mean similarity for each patient across the set of trajectories.

### Data and material approval

This study has been approved by the Danish Data Protection Agency (ref: SUND-2016-83) and the Danish Health Authority (refs: FSEID-00001627, FSEID-00003092, and FSEID-00003096).

### Reporting summary

Further information on research design is available in the Nature Research Reporting Summary linked to this article.

## Supplementary information

Supplementary Information

Reporting Summary

## Data Availability

Aggregate data created in this study are available from the corresponding author on reasonable request. Permission to access and analyse the underlying person-sensitive data can be obtained following approval from the Danish Data Protection Agency and the Danish Health Authority. Due to privacy concerns the data has to be analysed in closed analysis environments.

## Code availability

In order to analyse the data, the following software tool was used: R v. 3.6.1.
