# Supplementary material for: Optimizing drug selection from a prescription trajectory of one patient
Source: NPJ Digit Med. 2021 Oct 20;4:150. doi: 10.1038/s41746-021-00522-4 (PMC8528868; doi:10.1038/s41746-021-00522-4)
Supplement: Supplementary file 1 — Supplementary Information [file 41746_2021_522_MOESM1_ESM.pdf]

Optimizing drug selection from a prescription trajectory of one patient

Alejandro Aguayo-Orozco[1,2], Amalie Dahl Haue[1,3], Isabella Friis Jørgensen[1], David Westergaard[1,2,4], Pope Lloyd Moseley[1], Laust Hvas Mortensen[2, 5,*], Søren Brunak[1, *]

[1] Novo Nordisk Foundation Center for Protein Research, Faculty of Health and Medical Sciences, University of Copenhagen, DK-2200 Copenhagen, Denmark

[2] Statistics Denmark, DK-2100 Copenhagen, Denmark

[3] The Heart Center, Righospitalet, Copenhagen University Hospital, Copenhagen, Denmark

[4] Department of Obstetrics and Gynaecology, Amager Hvidovre Hospital, Copenhagen University Hospital, Hvidovre, Denmark

[5] Section of Epidemiology, Department of Public Health, University of Copenhagen, DK-1014 Copenhagen, Denmark

* Corresponding authors: Prof Søren Brunak, Novo Nordisk Foundation Center for Protein Research, Faculty of Health and Medical Sciences, University of Copenhagen, Blegdamsvej, 2200 Copenhagen, Denmark, E-mail: soren.brunak@cpr.ku.dk, Phone: +45 35 32 50 26. Prof Laust Hvas Mortensen, Statistics Denmark, DK-2100 Copenhagen, Denmark, E-mail: lhm@dst.dk, Phone: +45 39 17 32 18

Supplementary material

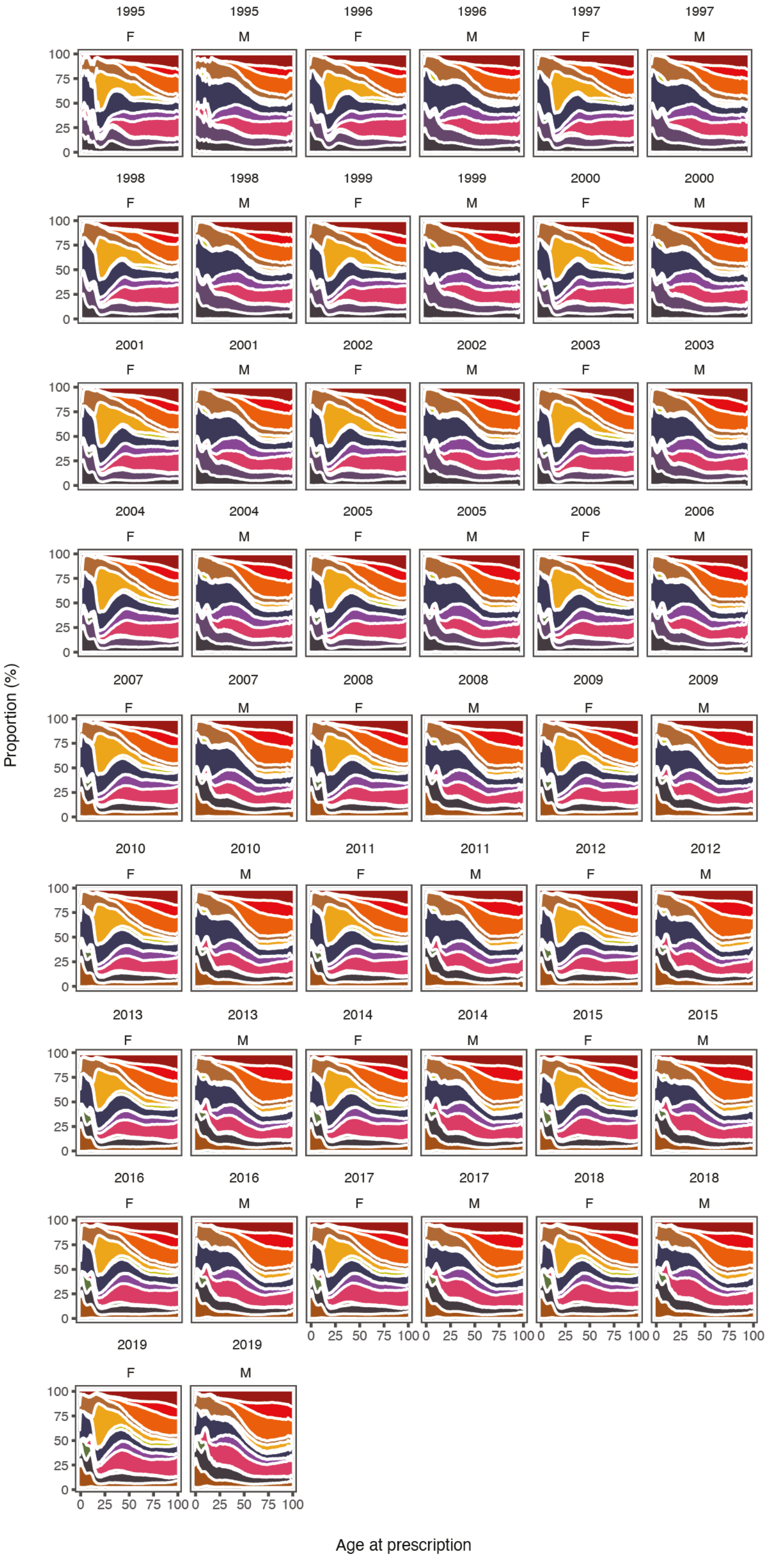

Supplementary figure 1 | Prescriptions redeemed at all Danish pharmacies in the period 1995-2019 stratified by the 14 anatomical ATC groups for males (left) and females (right) and by year of redemption. ATC main group prescription redemption separated for each year and sex in the study (period 1995-2019). It shows the proportion of different ATC groups by age (x-axis) for each year in the study period and separated for males and females. *Refer to Figure 1 for ATC group legend.

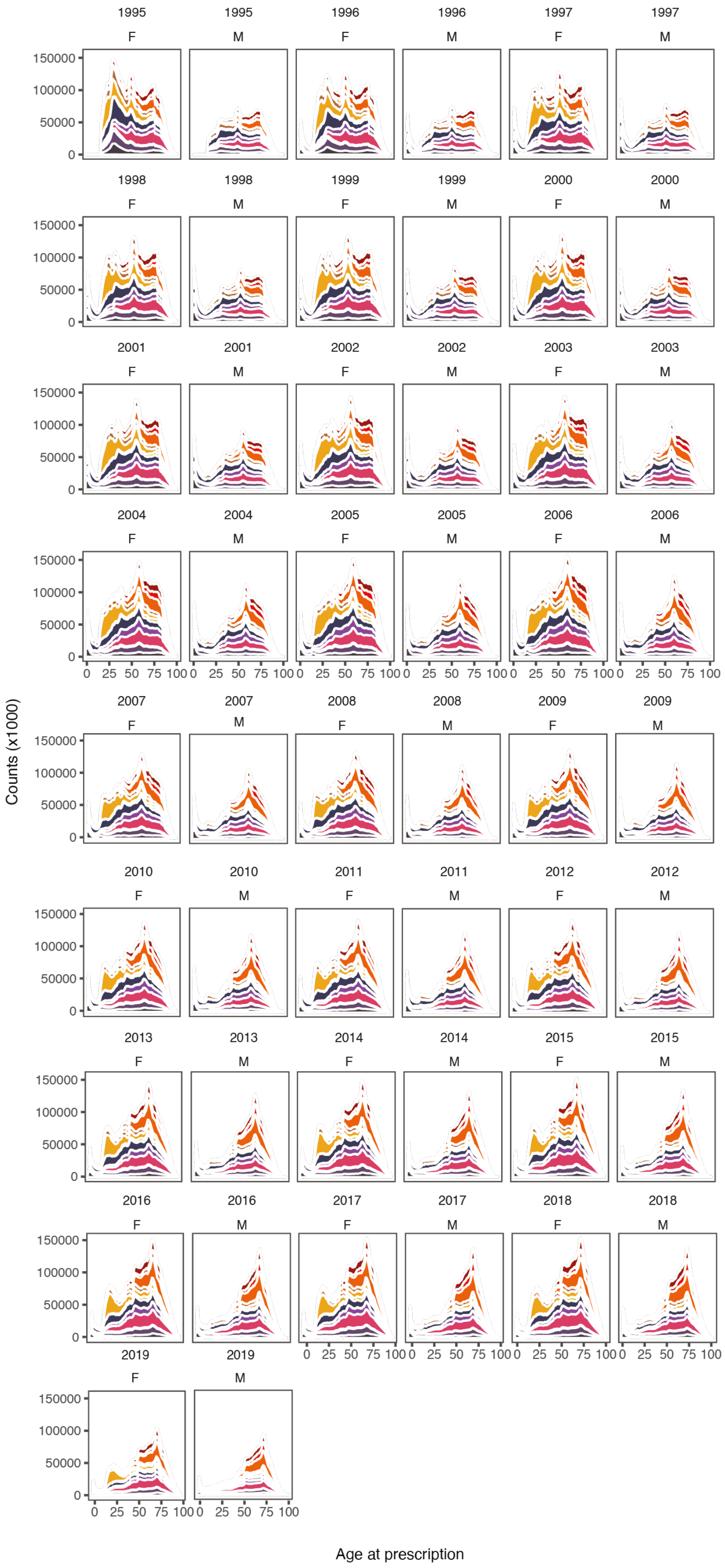

Supplementary figure 2 | Counts of prescriptions redeemed at all Danish pharmacies in the period 1995-2019 stratified by the 14 anatomical ATC groups for males (left) and

females (right) and by year of redemption. ATC main group prescription redemption separated for each year and sex in the study (period 1995-2019). It depicts the number of patients per 1,000 patients redeeming each ATC main group at different ages (x-axis) for each year in the study period and separated for males and females. *Refer to Figure 1 for ATC group legend.

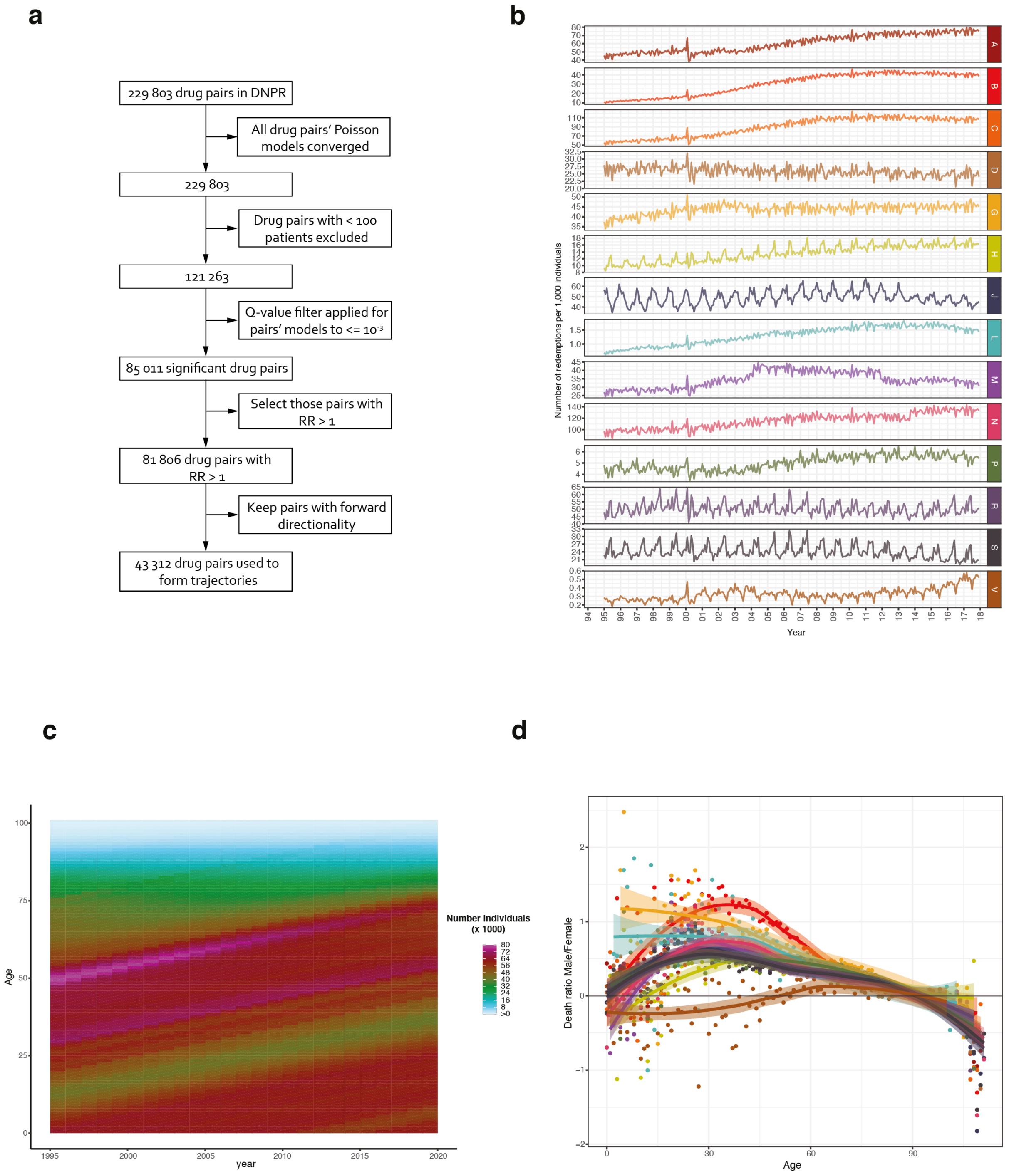

Supplementary figure 3 | Cohort description and workflow of drug pair inclusion. a, drug pair inclusion flow chart**. b,** seasonal graph for each ATC class, where prescription redemption per 1,000 patients displays seasonal patterns. **c**, Lexis diagram representing the population used in this study, including all the patients alive in the period 1995-2019, from the age 0 to 100. **d,** death ratio between male (up) and female (down) across all ages. Ratio is based on prescription redemption by each sex at each age separated by ATC class. Refer to Figure 1 for ATC class legend. *Refer to Figure 1 for ATC group legend.

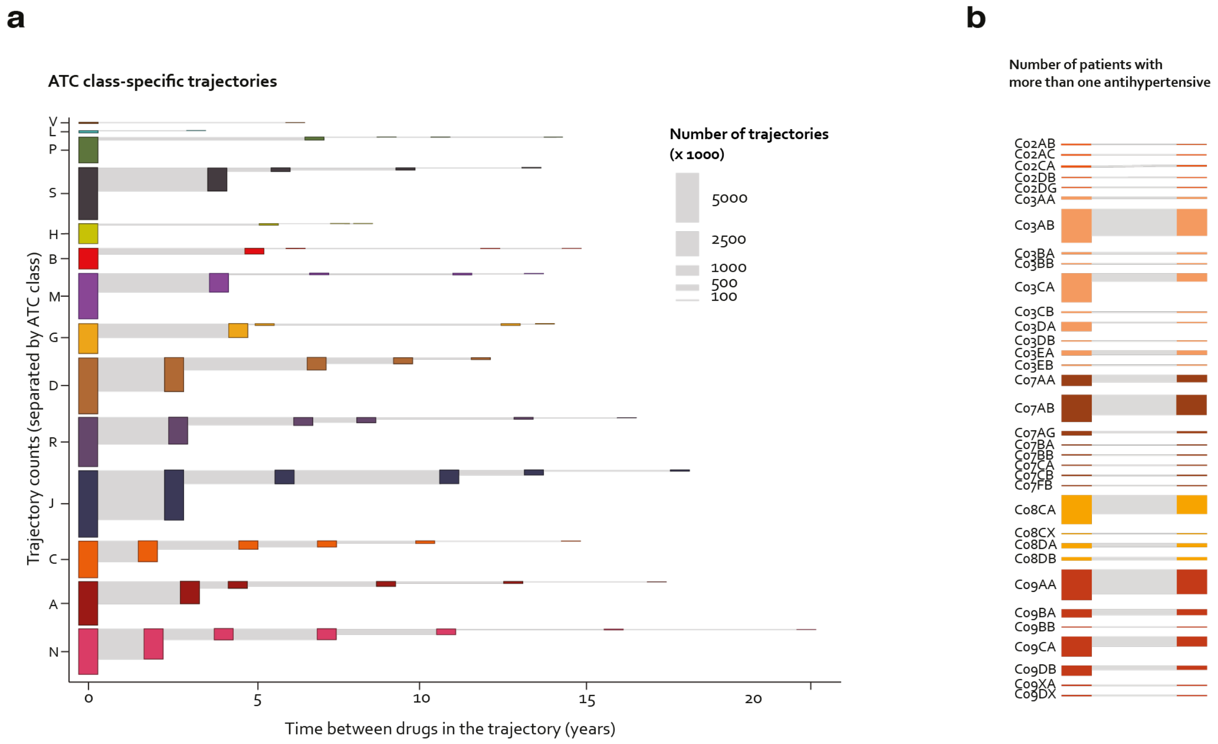

Supplementary figure 4 | Longitudinal trajectories formed by the same ATC group. a, trajectories formed by drugs from the same anatomical ATC group were included and then horizontally separated by average time between prescription redemptions. Each group of trajectories contains edges that separate the different prescription redemptions, whose height represent the number of trajectories that go from length 2 to length 3, to length N. Each group of trajectories is ordered from shortest to longest (top to bottom). *Refer to Figure 1 for ATC group legend. **b,** Number of individuals redeeming the subgroup indicated on the left side (node size) and how many of these patients redeem another drug used in hypertension (C02, C03, C07, C08, C09) posteriorly (ordered by chemical subgroup in ATC).

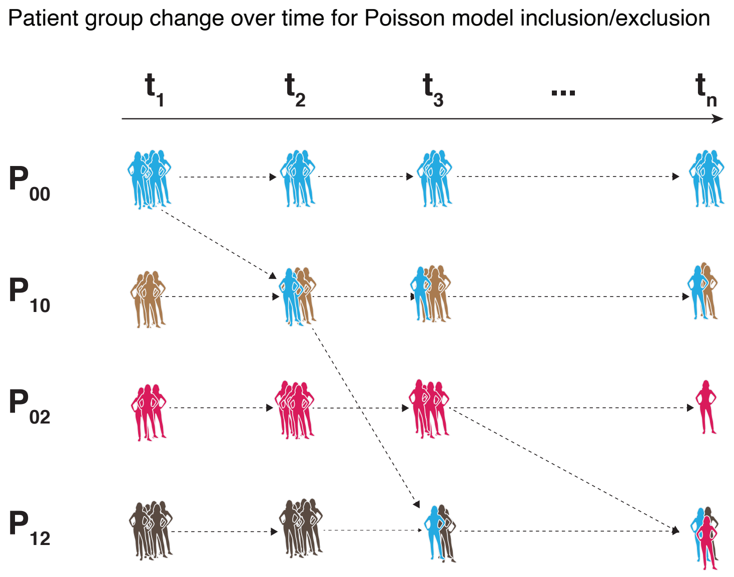

Year birth	Sex	time	P_{00}	P_{10}	P_{02}	P_{12}
1991	F	t_1	5	4	3	5
1991	F	t_2	4	4	5	5
1991	F	t_3	4	5	4	3
1991	F	t_n	4	3	1	3

Supplementary figure 5 | Individual grouping for Poisson modelling change over time. Each group of individuals, separated by sex and year of birth are dynamically allocated to different groups at each time point, depending on their prescription redemption at the time. That process is repeated for each stratum of the data (i.e., for each sex, year of birth and time of prescription), for each drug pair. As an example, women born in 1991 are used in the figure. They enter the study in 1995 (t_1) for prescription redemption pair (P1→P2). Some of these individuals redeemed both prescriptions at t_1, so they are directly counted in P_{12} (P_{12} = has redeemed P1 and P2; P_{10} = has redeemed P1 but not P2; P_{02} = has redeemed P2 but not P1; P_{00} = has redeemed neither P1, nor P2). This individual is then censored from the rest of the years in the model. Other individuals in the same stratum at t_1 will not have redeemed any of the drugs in the pairs under study, P1 or P2, hence they will be counted as P_{00}. At each time, the position of the different individuals might change, as they might have redeemed one or both prescriptions, or they might have left the study (emigration or death).

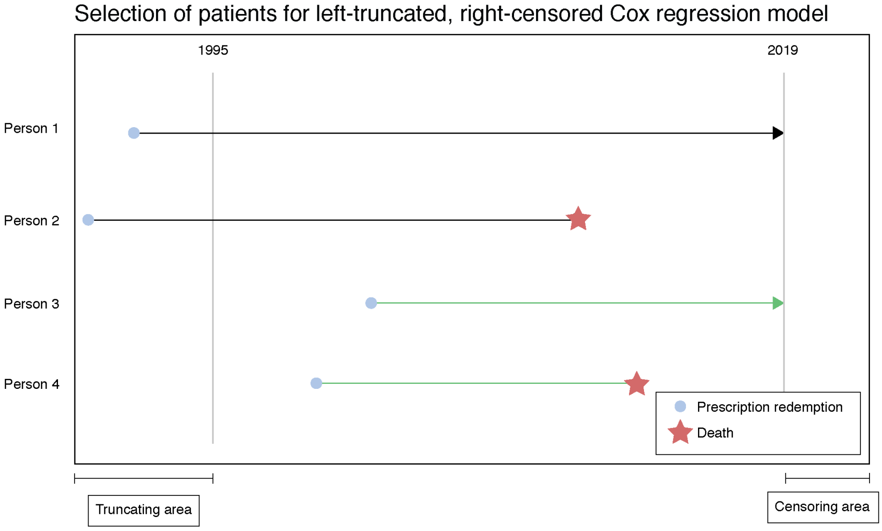

Supplementary figure 6 | Period of inclusion, 1995-2019, for Cox proportional hazard regression model cohort. Individuals who redeemed the prescription before 1995 are excluded from the cohort (left-truncated) and patients who leave the study, either due to end of window of study (2019), emigration or other are censored (right censored). The timeframe is the period for which prescription registry has information (1995-2019), leaving those patients who redeemed the prescription before the beginning of the registry truncated out of the model (Supplementary Fig. 2). Charlson Comorbidity Index (CCI)[20] was calculated using the ICD-10 coded disease data from the Danish National Patient Registry. Age, sex and CCI were added as covariates in the model and an interaction with them was included if the hazard proportionality assumption was violated – using a test based on Schoenfeld residuals with a p-value lower than 0.0001.

Supplementary table 1 | Cohort characteristics.

	Male	Female
Number of patients (%)	3 586 032 (49 %)	3 669 887 (51%)
Median age 1995, years	30	30
Mean age 1995, years	31·2	32·9
Range age 1995, years	0-106	0-110
Median age 2019, years	44	47
Mean age 2019, years	43·9	47·1
Range age 2019, years	0-110	0-110
**Prescription redemption**		
Median	11	17
Mean	13·9	19·1
SD	10·7	13·4
Range	1-164	1-129

Supplementary table 2 | Prescription distribution over the Anatomical Therapeutic Chemical Classification System.

	Male					Female				
**Age**	**0-14**	**15-44**	**45-64**	**65-84**	**>85**	**0-14**	**15-44**	**45-64**	**65-84**	**>85**
**ATC mean (SD)**										
A	1·3 (0·6)	1·7 (1·1)	2·1 (1·7)	2·5 (1·9)	2·3 (1·6)	1·3 (0·6)	2·0 (1·4)	2·3 (1·8)	2·9 (2·1)	2·7 (1·7)
B	1·0 (0·3)	1·1 (0·4)	1·2 (0·5)	1·4 (0·7)	1·4 (0·6)	1·0 (0·2)	1·2 (0·5)	1·2 (0·5)	1·4 (0·7)	1·4 (0·6)
C	1·3 (0·7)	1·5 (1·1)	2·8 (2·1)	3·2 (2·2)	2·2 (1·5)	1·3 (0·7)	1·5 (1·0)	2·6 (1·9)	3·2 (2·3)	2·4 (1·6)
D	1·9 (1·3)	2·3 (1·7)	2·2 (1·6)	2·2 (1·6)	1·9 (1·3)	2·0 (1·3)	2·7 (2·0)	2·3 (1·6)	2·3 (1·6)	1·9 (1·3)
G	1·0 (0·2)	1·1 (0·3)	1·2 (0·5)	1·4 (0·6)	1·2 (0·5)	1·1 (0·3)	2·1 (1·3)	1·7 (1·0)	1·4 (0·7)	1·2 (0·5)
H	1·0 (0·1)	1·0 (0·2)	1·0 (0·2)	1·1 (0·3)	1·0 (0·2)	1·0 (0·2)	1·1 (0·4)	1·1 (0·4)	1·1 (0·4)	1·0 (0·3)
J	1·0 (1·0)	2·2 (1·3)	2·1 (1·4)	2·5 (1·6)	2·2 (1·4)	1·9 (0·2)	3·3 (1·9)	2·6 (1·7)	2·7 (1·8)	2·4 (1·6)
L	1·1 (0·3)	1·1 (0·3)	1·1 (0·3)	1·1 (0·3)	1·0 (1·8)	1·1 (0·2)	1·0 (0·2)	1·1 (0·3)	1·0 (0·2)	1·0 (0·2)
M	1·1 (0·3)	1·5 (0·7)	1·7 (0·9)	1·7 (0·9)	1·4 (0·7)	1·1 (0·3)	1·6 (0·9)	1·8 (1·1)	1·9 (1·2)	1·5 (0·8)
N	1·3 (0·8)	2·5 (2·3)	2·7 (2·3)	3·0 (2·3)	2·8 (1·9)	1·3 (0·8)	2·7 (2·4)	3·0 (2·5)	3·4 (2·5)	3·1 (2·1)
P	1·0 (0·2)	1·2 (0·5)	1·1 (0·4)	1·1 (0·3)	1·0 (0·2)	1·0 (0·2)	1·3 (0·5)	1·2 (0·5)	1·1 (0·3)	1·0 (0·2)
R	2·0 (1·3)	2·0 (1·3)	1·9 (1·3)	2·2 (1·7)	1·7 (1·1)	1·8 (1·2)	2·4 (1·7)	2·2 (1·7)	2·3 (1·8)	1·7 (1·5)
S	1·5 (0·8)	1·6 (1·0)	1·7 (1·1)	2·1 (1·5)	1·7 (1·1)	1·4 (0·8)	1·7 (1·0)	1·8 (1·3)	2·2 (1·6)	1·9 (1·3)
V	1·0 (0·1)	1·0 (0·1)	1·0 (0·1)	1·0 (0·1)	1·0 (0·2)	1·0 (0·1)	1·0 (0·1)	1·0 (0·1)	1·0 (0·1)	1·0 (0·1)
**Range**										
A	1-13	1-23	1-21	1-21	1-14	1-18	1-23	1-22	1-21	1-18
B	1-6	1-7	1-7	1-7	1-6	1-6	1-8	1-8	1-8	1-6
C	1-9	1-19	1-20	1-21	1-15	1-9	1-18	1-20	1-21	1-15
D	1-16	1-21	1-21	1-19	1-15	1-18	1-21	1-19	1-18	1-15
G	1-4	1-7	1-11	1-7	1-5	1-5	1-14	1-11	1-9	1-7
H	1-4	1-5	1-5	1-5	1-4	1-5	1-7	1-6	1-5	1-4
J	1-17	1-20	1-16	1-14	1-11	1-17	1-21	1-16	1-15	1-13
L	1-4	1-5	1-6	1-5	1-3	1-5	1-5	1-5	1-5	1-4
M	1-6	1-9	1-11	1-10	1-7	1-6	1-10	1-11	1-11	1-9
N	1-19	1-33	1-34	1-27	1-18	1-14	1-36	1-30	1-28	1-19
P	1-5	1-6	1-7	1-5	1-3	1-5	1-7	1-6	1-5	1-4
R	1-14	1-17	1-17	1-17	1-13	1-14	1-19	1-19	1-18	1-13
S	1-13	1-16	1-16	1-17	1-13	1-13	1-17	1-16	1-15	1-14
V	1-2	1-3	1-3	1-3	1-2	1-2	1-3	1-3	1-2	1-2

Supplementary table 3 | Relative risk for pairs in figure 2

ATC 1	ATC 12	Number patients	RR	95% CI	P-value
N02AA	N02AB	138 110	2.76	2.74 – 4.13	2.70e-08
R03CC	R03BA	226 898	2.72	1.86 - 3.99	2.68e-07
C09AA	C09BA	164 680	2.79	1.93 - 4.03	5.22e-08
C07AB	B01AA	125 137	3.17	2.17 - 4.63	2.72e-09
C07AB	C01AA	103 043	3.55	2.31 - 5.47	9.07e-09
A03FA	N02AB	102 783	2.77	1.89 - 4.05	1.42e-07
N06AB	N05AF	116 605	2.86	2.02 - 4.0	4.11e-09
B01AC	C01AA	135 262	2.88	1.89 - 4.38	8.06e-07
N05BA	N02AG	118 223	2.83	2.09 - 3.83	1.83e-11
C09AA	C01DA	137 306	3.14	2.1 - 4.62	8.19e-09
J01CE	D07BB	195 989	2.81	2.19 - 3.61	5.51e-16
M01AB	N02AG	118 272	2.80	2.0 - 3.76	5.61e-12
M01AC	M01AB	121 302	2.73	2.08 - 3.58	3.70e-13
G03AA	J01EB	391 118	3.05	1.93 - 4.82	1.87e-06
J01EB	G01AF	189 887	3.28	2.33 - 4.63	1.19e-11
A02BA	N05BA	138 066	2.89	2.18 - 3.83	1.16e-13
M01AX	C09AA	105 249	2.88	2.06 - 4.04	8.30e-10
C10AA	C03CA	218 656	2.74	1.94 - 3.87	1.17e-08
G03AA	G01AF	279 484	3.98	2.42 - 6.55	5.27e-08
A02BA	A03FA	149 396	2.74	2.06 - 3.64	4.28e-12
D07BB	J01FA	131 233	3.13	2.42 - 4.04	1.93e-18
C10AA	A12BA	232 191	2.85	1.98 - 4.10	1.74e-08
S01GA	R06AE	113 185	2.74	2.07 - 3.61	1.27e-12
G03AA	D06BB	129 086	3.12	2.00 - 4.86	5.17e-07
M01AX	B01AC	113 398	2.98	2.11 - 4.21	5.70e-10
S01GA	S01GX	125 768	2.75	2.06 - 3.68	1.01e-11
C07AB	C03DA	121 672	2.90	1.99 - 4.23	3.44e-08
D07BB	D07AC	112 418	3.43	2.59 - 4.54	7.00e-18
A08AA	M01AB	185 846	2.97	2.28 - 3.87	7.04e-16
A02BA	R05FA	126 611	2.87	2.16 - 3.81	3.85e-13
G03AA	J02AC	414 591	2.82	1.76 - 4.53	1.66e-05
D07BB	S01AA	123 584	2.78	2.14 - 3.62	2.15e-14
M01AX	C08CA	111 180	2.95	2.07 - 4.20	2.48e-09
D07BB	D07AB	111 589	3.33	2.50 - 4.45	3.02e-16
D07BB	D01AC	120 234	3.26	2.47 - 4.29	4.61e-17
M01AX	C03CA	102 801	3.10	2.17 - 4.42	4.24e-10
C03AB	R05CB	102 408	2.78	1.92 - 4.03	5.53e-08
D07BB	A10BK	3 349	0.002	1.20e-03 - 3.15e-03	4.44e-142
D07CB	A10BK	1 538	2.27e-03	1.24e-03 - 4.17e-03	2.46e-86
M03BA	A10BK	1 264	2.32e-03	1.24e-03 - 4.35e-03	3.30e-80
S03BA	B01AF	1 505	3.24e-03	1.80e-03 - 5.82e-03	8.93e-82
S03BA	N05CH	1 789	3.25e-03	2.04e-03 - 5.19e-03	1.03e-127
G03CB	B01AF	6 461	3.39e-03	1.74e-03 - 6.62e-03	2.50e-62
S03BA	N05CH	1 789	3.25e-03	2.04e-03 - 5.19e-03	1.03e-127
G03CB	B01AF	6 461	3.39e-03	1.74e-03 - 6.62e-03	2.50e-62
G03CB	N05CH	6 023	3.71e-03	1.91e-03 - 7.18e-03	9.73e-62
C03CB	A10BH	449	6.49e-01	2.74e-01 - 1.53	3.24e-01
G03CB	R02AX	1 121	4.01e-03	2.02e-03 - 7.94e-03	2.24e-56
B01AF	N06DX	1 344	4.15e-01	3.74e-01 - 4.60e-01	1.38e-62
A11EA	G03FA	1 009	1.06e+01	8.51 – 13.1	6.12e-102
G01AG	S01CA	1 696	1.06e+01	7.10 - 15.7	3.04e-31
B03AE	A06AD	1 503	1.06e+01	6.51 – 17.1	1.40e-21

S01KA	M01AH	1 012	1.08e+01	7.57 – 15.4	1.49e-39
A11EA	G04CA	1 052	1.09e+01	8.58 – 14.0	3.49e-82
A11EA	G03CB	1 030	1.10e+01	9.47 - 12.8	8.01e-217
S01KA	A02AA	1 081	1.12e+01	7.81 – 16.0	7.48e-40
S01KA	A06AD	1 745	1.15e+01	7.85 – 16.7	1.40e-36
A10BB	N02BE	97 371	1.60e+03	1.52e+03 - 1.68e+03	2.22e-308
A10BF	N02BE	3 717	1.66e+03	1.61e+03 - 1.72e+03	2.22e-308
A10BB	N02AX	68 483	1.67e+03	1.61e+03 - 1.75e+03	2.22e-308
A10BB	N03AF	4 148	1.70e+03	1.65e+03 - 1.75e+03	2.22e-308
A10BB	N02AJ	22 433	1.72e+03	1.67e+03 - 1.77e+03	2.22e-308
A10BB	N03AA	1 403	1.74e+03	1.68e+03 - 1.80e+03	2.22e-308
A10BB	N02AA	52 922	1.74e+03	1.68e+03 - 1.81e+03	2.22e-308
A10BB	N03AG	2 891	1.75e+03	1.69e+03 - 1.80e+03	2.22e-308
A10BB	N02AG	14 877	1.76e+03	1.67e+03 - 1.85e+03	2.22e-308
A10BB	N02AE	12 015	1.79e+03	1.72e+03 - 1.87e+03	2.22e-308
A10BB	N02AB	17 553	1.79e+03	1.71e+03 - 1.88e+03	2.22e-308
A10BF	N03AX	1 178	1.86e+03	1.82e+03 - 1.90e+03	2.22e-308
A10BH	A10BK	13 699	4.05e-01	3.24e-01 - 5.06e-01	2.34e-15
A10BD	A10BK	12 727	4.06e-01	3.15e-01 - 5.24e-01	3.95e-12
A10BJ	A10BK	13 156	4.19e-01	3.35e-01 - 5.23e-01	1.93e-14
A10BH	A10BJ	13 537	4.55e-01	3.73e-01 - 5.56e-01	1.34e-14
N06DX	N05AD	2 607	4.72e-01	3.90e-01 - 5.70e-01	8.22e-15
A10BH	A10BD	8 676	4.80e-01	3.94e-01 - 5.85e-01	4.16e-13
A10BD	A10BJ	11 035	5.03e-01	4.03e-01 - 6.28e-01	1.24e-09
B01AE	B01AF	11 838	5.04e-01	4.23e-01 - 6.00e-01	1.41e-14
A10BG	A10BH	1 436	5.25e-01	4.01e-01 - 68.6	2.31e-06
N06DX	N02AB	4 151	5.30e-01	4.48e-01 - 62.7	1.10e-13
A11EA	A01AB	2 882	9.20	6.88 - 12.3	1.93e-50

A11EA	A11CA	1 065	9.44	6.95 - 12.8	9.89e-47
G01AG	G03DA	1 351	9.57	6.64 – 13.8	8.72e-34
A11EA	A10BA	1 331	9.73	6.71 - 14.1	2.88e-33
S01KA	S01BC	1 116	9.89	6.56 – 14.91	6.24e-28
S01KA	S01XA	1 175	10.28	7.71 - 13.7	9.65e-57
A11EA	A12AX	1 857	10.5	7.97 – 13.85	2.62e-62
G01AG	G02BA	2 112	10.70	6.62 – 17.5	8.48e-22
S01KA	S01CA	2 092	10.8	7.11 – 16.4	8.13e-29
B03AE	B03BA	1 103	9.87	6.78 – 14.37	6.95e-33

Supplementary table 4 | Stratification of patients by prescription trajectories

	ACE treatment with no change	ARB treatment with no change	ACE treatment with change to ARB	ARB treatment with change to ACE
**Number of patients**	549 436 (49.3)	287 488 (25.8)	229 216 (20.6)	48 054 (4.3)
**Age, years**				
Mean (SD)	63.3 (13·9)	61.7 (13·3)	60.7 (12·3)	61.0 (13·2)
Median (IQR)	63.7 (20.0)	62.0 (18.9)	61.0 (17.4)	61.2 (18.9)
**Sex**				
Male	257 099 (46.8)	153 527 (53.4)	122 549 (53.5)	24 484 (51.0)
Female	292 337 (53.2)	133 961 (46.6)	106 667 (46.5)	23 570 (49.0)
**Charlson comorbidities**				
Myocardial infarction	74 244 (13.5)	14 962 (5.2)	23 253 (10.1)	5 485 (11.4)
Congestive heart failure	90 247(16.4)	13 696 (4.8)	26 115 (11.4)	6 668 (13.9)
Peripheral vascular diseases	59 155 (10.7)	18 643 (6.5)	20 914 (9.1)	5 666 (11.8)
Cerebrovascular disease	102 441 (18.6)	37 758 (13.1)	35 109 (15.3)	9 894 (20.6)
Dementia	29 033 (4.9)	8 387 (2.9)	6 181 (2.7)	2 452 (5.1)
Chronic obstructive pulmonary disease	77 099 (14.0)	31 317 (10.9)	28 892 (12.6)	7 029 (14.6)
Rheumatoid disease	21 897 (3.9)	10 172 (3·5)	9 500 (4.1)	2 135 (4.4)
Peptic ulcer disease	36 803 (6.7)	13 572 (4.7)	12 545 (5.4)	3 315 (6.9)
Mild liver disease	12 827 (2.3)	5 527 (1.9)	4 739 (2.0)	1 175 (2.4)
Diabetes without complications	90 186 (16.4)	27 143 (9.4)	37 033 (16.1)	8 391 (17·4)
Diabetes with complications	31 608 (5·7)	7 560 (2.6)	12 894 (5.6)	2 806 (5.8)
Hemiplegia or paraplegia	3 633 (0.7)	1 433 (0.5)	1 064 (0·4)	298 (0.6)
Renal disease	29 369 (5.3)	8 112 (2.8)	11 624 (5.1)	3 236 (6.7)
Cancer	102 249 (18.6)	44 873 (15.6)	39 402 (17.1)	9 362 (19.5)
Moderate or severe liver disease	4 272 (0·7)	1 438 (0.5)	1 174 (0·5)	341 (0.7)
Metastatic solid tumour	20 741 (3.7)	8 219 (2.8)	6 841 (2·9)	1 774 (3.7)
AIDS/HIV	295 (0·05)	109 (0.04)	85 (0·03)	23 (0·04)

Data are n (%) unless otherwise specified

Supplementary table 5 | Population continent of origin

	Percentage
Europe	92.50
Asia	2.93
Middle East	1.94
Africa	1.24
North America	0.81
South and Central America	0.39
Oceania	0.16
Not stated	0.03
