# Supplementary material for: Optimizing drug selection from a prescription trajectory of one patient
Source: NPJ Digit Med. 2021 Oct 20;4:150. doi: 10.1038/s41746-021-00522-4 (PMC8528868; doi:10.1038/s41746-021-00522-4)
Supplement: Supplementary file 2 — Reporting Summary [file 41746_2021_522_MOESM2_ESM.pdf]

nature portfolio

Corresponding author(s): Søren Brunak and Laust Hvas Mortensen

Last updated by author(s): Aug 17, 2021

Reporting Summary

Nature Portfolio wishes to improve the reproducibility of the work that we publish. This form provides structure for consistency and transparency in reporting. For further information on Nature Portfolio policies, see our Editorial Policies and the Editorial Policy Checklist.

Statistics

For all statistical analyses, confirm that the following items are present in the figure legend, table legend, main text, or Methods section.

n/a	Confirmed	
☒	☐	The exact sample size (n) for each experimental group/condition, given as a discrete number and unit of measurement
☒	☐	A statement on whether measurements were taken from distinct samples or whether the same sample was measured repeatedly
☐	☒	The statistical test(s) used AND whether they are one- or two-sided<br>*Only common tests should be described solely by name; describe more complex techniques in the Methods section.*
☐	☒	A description of all covariates tested
☐	☒	A description of any assumptions or corrections, such as tests of normality and adjustment for multiple comparisons
☒	☐	A full description of the statistical parameters including central tendency (e.g. means) or other basic estimates (e.g. regression coefficient) AND variation (e.g. standard deviation) or associated estimates of uncertainty (e.g. confidence intervals)
☒	☐	For null hypothesis testing, the test statistic (e.g. F, t, r) with confidence intervals, effect sizes, degrees of freedom and P value noted<br>*Give P values as exact values whenever suitable.*
☒	☐	For Bayesian analysis, information on the choice of priors and Markov chain Monte Carlo settings
☒	☐	For hierarchical and complex designs, identification of the appropriate level for tests and full reporting of outcomes
☐	☒	Estimates of effect sizes (e.g. Cohen's d, Pearson's r), indicating how they were calculated

Our web collection on statistics for biologists contains articles on many of the points above.

Software and code

Policy information about availability of computer code

Data collection	n/a
Data analysis	In order to analyse the data, the following software tool was used: R v. 3.6.1.

For manuscripts utilizing custom algorithms or software that are central to the research but not yet described in published literature, software must be made available to editors and reviewers. We strongly encourage code deposition in a community repository (e.g. GitHub). See the Nature Portfolio guidelines for submitting code & software for further information.

Data

Policy information about availability of data

All manuscripts must include a data availability statement. This statement should provide the following information, where applicable:

- Accession codes, unique identifiers, or web links for publicly available datasets
- A description of any restrictions on data availability
- For clinical datasets or third party data, please ensure that the statement adheres to our policy

Permission to access and analyse the underlying person-sensitive data can be obtained following approval from the Danish Data Protection Agency and the Danish Health Authority. Due to privacy concerns the data has to be analysed in closed analysis environments. This study has been approved by the Danish Data Protection Agency (ref: SUND-2016-83) and the Danish Health Authority (refs: FSEID-00001627, FSEID-00003092, and FSEID-00003096)

Field-specific reporting

Please select the one below that is the best fit for your research. If you are not sure, read the appropriate sections before making your selection.

☒ Life sciences ☐ Behavioural & social sciences ☐ Ecological, evolutionary & environmental sciences

For a reference copy of the document with all sections, see nature.com/documents/nr-reporting-summary-flat.pdf

Life sciences study design

All studies must disclose on these points even when the disclosure is negative.

Sample size	The sample size is 7,873,901 individuals, determined by the amount of individuals alive and at risk of redeeming any prescription between January 1st 1995 and June 30th 2019.
Data exclusions	No exclusion criteria were applied
Replication	The UK Biobank self-reporte medication data was used to replicated patient stratification and survival analysis results on the provided example with renin-angiotensin system prescriptions.
Randomization	Individuals were grouped based on whether they had redeeming prescription (P1) and/or prescription 2 (P2) or not, sex, age and calendar year at prescription redemption.
Blinding	Blinding was not required as it was a population-wide analysis using register data.

Reporting for specific materials, systems and methods

We require information from authors about some types of materials, experimental systems and methods used in many studies. Here, indicate whether each material, system or method listed is relevant to your study. If you are not sure if a list item applies to your research, read the appropriate section before selecting a response.

Materials & experimental systems

n/a	Involved in the study	
☒	☐	Antibodies
☒	☐	Eukaryotic cell lines
☒	☐	Palaeontology and archaeology
☒	☐	Animals and other organisms
☒	☐	Human research participants
☒	☐	Clinical data
☒	☐	Dual use research of concern

Methods

n/a	Involved in the study	
☒	☐	ChIP-seq
☒	☐	Flow cytometry
☒	☐	MRI-based neuroimaging
